# Clinical Characteristics of Visual Dysfunction in Carbon Monoxide Poisoning Patients

**DOI:** 10.1155/2020/9537360

**Published:** 2020-09-22

**Authors:** Wei-Kang Bi, Jin-Lin Wang, Xu-Dong Zhou, Ze-Kun Li, Wen-Wen Jiang, Shao-Bin Zhang, Yong Zou, Ming-Jun Bi, Qin Li

**Affiliations:** ^1^Weifang Medical University, Weifang, Shandong 261042, China; ^2^Department of Integration of Chinese and Western Medicine, Yantai Yuhuangding Hospital Affiliated to Qingdao University, Yantai, Shandong 264000, China; ^3^Emergency Center, Yuhuangding Hospital Affiliated to Qingdao University, Yantai, Shandong 264000, China; ^4^Shandong Wendeng Osteopathic Hospital, Yantai, Shandong 264000, China; ^5^Centre of Integrated Chinese and Western Medicine, School of Basic Medicine, Qingdao University, Qingdao, Shandong 266071, China; ^6^Weifang Eye Hospital, Weifang, Shandong 261000, China

## Abstract

**Purpose:**

The aim of the present study was to analyze the clinical characteristics of visual dysfunction in patients with carbon monoxide (CO) poisoning.

**Methods:**

A total of 436 patients with CO poisoning were enrolled in our hospital from October 2012 to December 2018, including 193 patients with moderate poisoning (MP group), 165 with severe poisoning (SP group), and 78 with delayed encephalopathy (DE group). The clinical characteristics of visual dysfunction in patients with CO poisoning were analyzed through the collection of medical history, regular physical examination, brain magnetic resonance imaging (MRI), ophthalmological examination, the National Eye Institute Visual Function Questionnaire (NEI-VFQ), and its influencing factors.

**Results:**

Some patients in the three groups had visual dysfunction. The main ocular symptoms were local pain, eye movement disorder, and visual field defect. The key pathological factors were keratopathy, retinal nerve cell damage, optic nerve damage, retinal vascular disease, macular disease, and occipital visual center damage. The clinical symptoms of visual dysfunction after CO poisoning lasted for a long time (>12 months) and were not completely consistent with the positive results of the ophthalmological examination. A few sequelae of ophthalmology were still left after the help of medicine.

**Conclusion:**

The incidence of visual dysfunction in patients with CO poisoning was high, the clinical symptoms were rich and diverse, the duration of disease was long, and the prognosis was poor. Thus, the relevant ophthalmological examination and intervention treatment should be perfected as soon as possible.

## 1. Introduction

Carbon monoxide (CO) is the most common asphyxiating gas that causes toxic death in life and work. Once poisoned, it can lead to multiple system dysfunction in patients, among which brain and myocardial damages are the most serious and become the main death factors in the acute phase [1, 2]. Despite the improvement of the clinical performance and the recovery of consciousness in acute CO poisoning patients, some cases still appear as series of encephalopathy symptoms again within 2–60 days of “false recovery period.” The latter is called the delayed encephalopathy after carbon monoxide poisoning (DEACMP) with high death rate and disability rate and seriously affects the life quality of patients and their families [3]. Actually, the visual dysfunction induced by CO poisoning is also common in clinical practice; especially, serious visual function damage will occur in severe poisoning patients [4, 5], even blindness, and the prognosis is particularly poor. At present, scholars around the world are mainly focused on the molecular mechanisms of the central nervous system and cardiovascular system caused by the toxicity after CO exposure. The evidence about the causes and specific mechanisms of visual dysfunction after CO poisoning is obviously insufficient in clinics and experiments for large-scale research. Therefore, based on the clinical cases, the present study aims to analyze the clinical and pathological characteristics of visual dysfunction in CO poisoning patients so as to provide a basis for the clinical treatment and prognosis.

## 2. Clinical Information and Methods

### 2.1. General Information

A total of 436 patients with CO poisoning were admitted in Yantai Yuhuangding Hospital Affiliated to Qingdao University from October 2012 to December 2018 in the present study and divided into three groups: a moderate poisoning group (MP group, *n* = 193), a severe poisoning group (SP group, *n* = 165), and a delayed encephalopathy group (DE group, *n* = 78) according to the clinical characteristics and severity of the disease. All candidates met the diagnostic criteria for the acute CO poisoning of occupational diseases and the delayed encephalopathy of carbon monoxide poisoning (DEACMP) [6]. Patients with high blood carboxyhemoglobin concentration (30%∼49%) and mild to moderate coma after poisoning were enrolled in the moderate poisoning group, while those with deep coma or decancephalization, obvious cerebral edema, shock, severe myocardial damage, pulmonary edema, respiratory failure, and higher blood carboxyhemoglobin concentration (≥50%) were selected in the severe poisoning group. All patients and their families signed medical informed consents, actively cooperated with the treatment, and completed all follow-up plans. Exclusion criteria were as follows: (1) patients younger than 18 or older than 75; (2) patients with cerebrovascular disease and its sequelae, cirrhosis, kidney disease, cardiovascular, or other chronic diseases with organ dysfunction at the end period; (3) patients with genetic and congenital disease; (4) patients with malignant tumor, primary immune dysfunction, or received immunosuppressive therapy recently; (5) patients with a medical history of ophthalmic diseases before; (6) cognitive impairment, midway death, loss of contact, or withdrawal from the follow-up plan.

The general information of all patients was collected by two professionals, including name, age, gender, body mass index, duration of CO poisoning, concentration of carboxyhemoglobin (COHb) in arterial blood, coma time, course of disease, clinical symptoms of ophthalmology, clinical signs, and results of special examination. The data were summarized and analyzed by medical statistics. There was no statistical difference in general demographic characteristics among the three groups (Table 1). All patients were given conventional hyperbaric oxygen treatment and internal medical therapy after admission. According to the results of clinical manifestations and visual function examination, the patients with visual dysfunction were given corresponding treatment. Mannitol was given to reduce intracranial pressure and papilledema in cases with intracranial hypertension and papilledema; vasodilators were used to relieve cerebral and local vasospasm, improve microcirculation, and increase blood flow perfusion of eye tissue; neurotrophic agents and multivitamins were used to promote the recovery of brain tissue and optic nerve function. Further, levofloxacin, 0.1% sodium hyaluronate and fluorometholone, recombinant bovine basic fibroblast growth factor, and other eye drops were given to the patients with keratopathy; brimonidine tartrate and brinzolamide eye drops were given to the patients with glaucoma; iodized lecithin tablets were given to the patients with vitreous lesions.

### 2.2. Visual Acuity, Visual Field, and Fundus Examination

Patients in both the moderate poisoning group and the severe poisoning group had disturbance of consciousness to some extent when they were admitted to the hospital. Therefore, they should be treated actively in the intensive care unit immediately after admission. The first ophthalmic examination should be carried out when their consciousness returned to normal and could cooperate with the examination on the second or third day after admission generally. Then the ophthalmic examinations were followed up to observe the changes of ocular clinical manifestations on 7 days, 1 month, and 2 months after poisoning. The visual acuity test was performed by the standard vision chart. The visual field test was carried out by 640 Humphrey computer automatic visual field analyses (Carl Zeiss Company, USA) according to 30-2 static full threshold and 60-2 peripheral program under the condition of the natural pupil. The visual field examination was done with the best-corrected visual acuity under the natural pupil. The results were considered to be reliable if it met the following conditions: (1) fixation loss < 20%; (2) short-term fluctuation < 2 db; (3) false-positive and false-negative rate < 30%; and (4) patients with corresponding arcuate visual field defect in both 30-2 and 60-2. Fundus examination was performed with fundus glasses.

### 2.3. Flash Visual Evoked Potential (FVEP) Examination

The latencies and amplitudes of VEP waves in each patient were detected by the visual electrophysiological instrument Roland RETIPort21 made in Germany. The whole detection process was carried out in a quiet dark room. The patients sat in front of the ganzfeld stimulator with full field of vision and kept the mind and muscles relaxed and focused completely. During the detection, one eye gazed at the midpoint of the screen and the opposite should be tightly covered to ensure not to be stimulated by light. Then the eyes were examined alternately. There was no need to dilate the pupils. If the pupil was too big or too small before the examination, it should be marked. Simultaneously, it should be completely corrected before detection if there was ametropia.

### 2.4. Brain MRI

The brain scanning was performed with 1.5 T magnetic resonance imaging (MRI) of Singa HDe in the GE company and an 8-channel phase-controlled head coil. Each patient underwent both conventional MRI and STIR scan sequence. The specific parameters of MRI were set as shown in Table 2.

### 2.5. Efficacy Evaluation

The complete recovery rate, improvement rate, nonhealing, and sequelae rate were analyzed at 2 months after poisoning, and expressed as follows: the complete recovery rate (or improvement rate or nonhealing and sequelae rate) = number of patients with complete recovery (or improvement or nonhealing and sequelae)/the total number of patients receiving regular ophthalmic treatment × 100%. The sequelae and clinical features were evaluated again in the 12 months.

At the same time, the life quality of the patients after treatment was investigated at 2 months after poisoning using the National Eye Institute Visual Function Questionnaire-25 (NEI-VFQ-25) developed by the National Institute of Ophthalmology. The questionnaire has been widely used in many ophthalmic diseases and has strong reliability and accuracy [7–9]. It consists of 12 dimensions and 25 items. Each item is scored at 6 levels, including 100, 75, 50, 25, 0 points, and “no response.” The score of each dimension is the sum of the corresponding items. The higher the score is, the better the dimension is. The total score is expressed as the sum of all items. Similarly, the higher the total score is, the better the visual function-related quality of life is. Cronbach's alpha reliability coefficient of the scale is 0.95, and the correlation coefficient of the content validity of each item is higher than 0.4, indicating that the content validity of the scale is quite fair. Cronbach's alpha coefficient of the scale is 0.788 based on the preinvestigation.

### 2.6. Statistical Analysis

SPSS 20.0 was used in this study. The measurement data were expressed as mean ± standard deviation. The analysis of variance was used to compare the differences between multiple groups, and the Newman–Keuls test was to analyze the comparison of the differences between any two groups. The counting data were expressed as percentage. Kruskal–Wallis *H* was used to analyze the differences among the three groups, and then the Nemenyi test was to compare the differences between any two groups. *P* value <0.05 was considered as a significant difference.

## 3. Results

### 3.1. Clinical Manifestations of Ophthalmology in Different Groups

Among the three groups, there were a few patients with different degrees of ophthalmic clinical symptoms, including eye discomfort, eye swelling, eye pain, photophobia, tears, blurred vision, visual field defect, diplopia, and blindness. The main pathological changes were roughly divided into several types as follows according to lesion tissue: keratopathy, glaucoma, optic neuropathy, retinal vascular disease, oculomotor neuropathy, vitreous disease, macular disease, intracranial disease, trauma, and hysteria, among which keratopathy, optic neuropathy, retinal vascular disease, macular disease, and intracranial diseases were more common in clinical practice. The special examination of ophthalmology showed cornea inflammation, corneal ulcer, corneal edema, elevated intraocular pressure, tortuous dilation of fundus vein, segmental filling, unclear boundary of optic disc, diffused or localized edema of retina, papilledema, and retrobulbar optic neuritis. The main manifestation of macular disease was cystoid macular edema with unclear light reflection of central fovea of macular. The clinical features of vascular retinopathy were segmental filling, stenosis or obstruction of retinal artery, vein embolism, hemorrhage, and peripheral exudation. There was no statistical difference among the three groups (Table 3, *P* > 0.05).

### 3.2. Consistency Comparison between the Complaint Symptom and the Ophthalmic Examination

Although a small number of patients in the three groups had clinical symptoms and/or ophthalmic examination abnormalities, in fact, not all patients with the clinical symptoms showed the obvious abnormalities in the ophthalmic examination, and not all patients with the ophthalmic examination abnormalities had the typical clinical symptoms yet. That is to say, the clinical symptoms of patients were not completely consistent with their ophthalmic examination. Even though they had serious examination abnormalities, some patients lacked typical clinical symptoms (Table 4). Therefore, after CO poisoning, it is necessary to improve the ophthalmic examination as soon as possible, so as to find the lesion focus and intervene as early as possible.

### 3.3. The Analysis of Flash Visual Evoked Potential (FVEP) Examination in Three Groups

In the present study, FVEP was used to evaluate the visual function of patients. The latency and amplitude of P1 wave in FVEP were selected as the detection criteria: slight latency delay of P1-wave and/or decrease of amplitude <30% were diagnosed as mild abnormality, moderate latency delay of P1 and/or amplitude reduction of P1 from 30% to 70% were considered as moderate abnormality, and severe delay and/or amplitude reduction of P1 > 70% were severe abnormality. We found that there were abnormal results of FVEP in some patients of the three groups, mainly manifested as prolonged P1 latency and decreased amplitude. Among them, patients in the MP group had mild abnormal P1 wave latency and amplitude, while those in the DE group had severe abnormal P1 wave latency and amplitude. However, there was no significant difference among the three groups (Table 5).

### 3.4. Analysis of Clinical Symptoms, Ophthalmic Examination, and Brain MRI in Three Groups

Some of the patients in the three groups had visual dysfunction; however, the complaint symptom was often not completely consistent with the results of ophthalmic examination. The number of patients with abnormal ophthalmic examination was significantly more than those with complaints (Table 6). In addition to complaint symptoms and ophthalmic examination, brain MRI scan was of great significance in the diagnosis of acute brain injury and delayed encephalopathy caused by CO poisoning. Nevertheless, in the acute stage of CO poisoning, there was no characteristic manifestation in brain MRI. The symmetrical extensive or partial lesion with slightly longer *T*_1_ and slightly longer *T*_2_ signals was found in the bilateral parietal and occipital lobes, or temporal lobes, the boundary was unclear, and the cleavage of corresponding brain groove was narrow in typical MRI. We found the positive rate of typical MRI was not high, and there was no statistical difference between the MP and SP groups (*P* > 0.05, Table 6). Different from the image scan, in the MP and SP groups, the diffuse long *T*_1_ and long *T*_2_ signal changes in the DE group patients were often appeared in bilateral white matter and basal ganglia, along with slight edema of white matter, slight compression, and narrowing of the ventricular system, and similar changes also could be detected in bilateral basal ganglia. The cerebellopontine and optic nerve could be damaged because of the CO toxicity, too. Neither obvious abnormality was found in the cerebral cortex, nor enhancement image in the enhanced MRI series. The positive rate of MRI in the DE group was significantly higher than that in the MP group and SP group (*P* < 0.05). Actually, the result of brain MRI was not completely consistent with the abnormal severity of complaint symptoms and ophthalmic examination.

### 3.5. Comparison of the Therapeutic Effect in Each Group after Normal Treatment

In the three groups, some patients had the chief complaints and the abnormality of ophthalmology examination. The main clinical characteristics were the diversity of clinical symptoms and the different duration of disease. Some patients were perfectly recovered from the ophthalmic diseases caused by CO poisoning within a few days, while others could not be completely relieved even after 12 months of regular treatment and a few left eye sequelae (Table 7). According to statistical analysis, the complete recovery rate and improvement rate of patients in MP and SP groups were significantly higher than those in the DE group (*P* < 0.05). Although the clinical manifestations of many patients in the DE group improved to some extent after regular treatment, the incidence of ophthalmic sequelae was obviously higher than that of the other two groups (*P* < 0.05).

### 3.6. Score Comparison of the National Eye Institute Visual Function Questionnaire-25 (NEI-VFQ-25) in the Three Groups in Two Months after Treatment

In the three groups, the scores of NEI-VFQ-25 in most of the patients were declined to some extent, mainly reflected in the general health, general vision, ocular pain, near activities, distance activities, social function, mental health, role difficulties, dependency, driving, color vision, peripheral vision, and other aspects. According to the statistical analysis, the subscales of NEI-VFQ-25 that were more sensitive to the decline of visual function-related quality of life caused by CO poisoning were general health, general vision, near activities, distance activities, social function, mental health, role difficulties, dependency, and peripheral vision. There were statistical differences among the three groups (Table 8, *P* < 0.05). The differences in social function, mental health, role difficulties, and dependency were more significant. The visual function-related quality of life in the MP group was better than that in the SP group and the DE group (*P* < 0.05), suggesting that once CO poisoning occurs, patients must go to the hospital for medical help, so as to find the lesion and intervene treatment as early as possible, delay the progress of the disease, prevent and treat complications and sequelae, and improve the visual function-related quality of life in patients.

## 4. Discussion

CO poisoning is very common in daily life and work and becomes a leading factor of accidental poisoning in many industrial countries [10, 11]. In the United States, the mortality rate induced by CO poisoning is the highest and results in more than 50% fatal poisonings at the stage of acute onset [12]. The central nervous system is mostly sensitive to hypoxia. The optic nerve and retina are the most important structural basis of visual function. As a part of nervous tissue, they are highly sensitive to hypoxia and often suffered CO toxicity. Our results showed that the clinical ocular manifestations after CO poisoning were various, including one-off, paroxysmal, or permanent visual impairment and even cortical blindness, which is consistent with the results of Levin et al. [13] and Chen et al. [14].

At present, the reason for visual dysfunction after CO poisoning was poorly understood. Most scholars thought that it might be related to the following factors: (1) After CO poisoning, it could cause hypoxia of the whole body organs and make the brain tissue and the whole body ATP synthesis disorder, Na^+^-K^+^ pump transport out of control, K^+^ overflow, and Na^+^ retention in cells, resulting in neurocyte edema (including the visual center of the occipital lobe, visual radiation, and visual cross of the visual pathway). (2) Anoxia caused by CO poisoning involves a wide range of organisms. Severe hypoxia causes cerebral vasospasm, followed by continuous vasodilation. The accumulation of acid metabolites increases vascular permeability, brain tissue interstitial edema and progressive increase of intracranial pressure result in the rise of central retinal vein pressure, and the outflow passage obstruction of tissue fluid along the optic nerve and the retardation of axoplasmic transportation finally lead to the passive hyperemia and edema of optic papilla. (3) Due to severe ischemia and hypoxia after CO poisoning, the damage of vascular endothelial cells in the brain and fundus leads to the roughness of intima, the increased aggregation and adhesiveness of platelet, and the release of a large number of vasoactive substances, as well as vasospasm. The latter induces leukocyte aggregation, adhesion, and infiltration into the intima of vessels, resulting in microthrombosis or local blood circulation disorder. In some severe cases, focal ischemic lesions, even necrosis, and punctate hemorrhage could be found in the cortex and basal ganglia. (4) Endogenous and exogenous CO play different roles in vivo. It has been demonstrated that endogenous CO can rapidly pass through the cell membrane, act on neurocytes in the form of autocrine and paracrine, and cause vasodilation in the brain and fundus through cyclic guanosine monophosphate (cGMP) signal transduction pathway [15, 16]. In addition, CO can also play different roles in vivo through hyperpolarization, peroxide, calcium dynamic conversion, and other mechanisms [17]. (5) The hypoxia of the retina after CO poisoning can make the vessel system of the retina itself produce transient or continuous spasm, change the permeability of vessel wall, then aggravate the edema of the retina, and even cause retinal hemorrhage. (6) CO poisoning can cause excessive oxidative stress mediated by leukocytes and produce a large number of oxygen free radicals. The resulting free radical cascade reaction can cause the dysfunction of the oxidation/antioxidant signal system in vivo and aggravate the apoptosis of neurons in the visual pathway. Furthermore, excessive oxidative stress and abnormal immunity may promote and worsen the demyelination of the central nervous system. (7) The formation of the myelin sheath of the optic nerve was asymmetric, and each part of the optic nerve had different dependence on the number of mitochondria and energy. After CO poisoning, the mitochondrial membrane potential of nerve cells decreased significantly, and the structure and function of mitochondria were damaged seriously [18], accompanied with the accumulation of oxidative stress, which was easy to lead to the selective degeneration of small optic nerve fibers.

Hyperbaric oxygen therapy has been considered as the most effective treatment for patients with acute CO poisoning. It can rapidly improve the partial pressure of blood oxygen and the content of tissue oxygen, rapidly reduce the concentration of COHb in the blood of patients, alleviate systemic hypoxia, and relieve the inhibition of biological oxidation caused by CO poisoning. Especially, it plays a crucial role in the repair of the occipital visual center and visual radiation. However, some studies have shown that the CO inhalation at low concentrations could cause the decrease in patients' dark adaptability, and this symptom always existed even when the concentration of COHb in blood was reduced to the normal range. It indicates that the visual dysfunction caused by acute CO poisoning will not disappear due to the discharge of CO in vivo or the rapid decrease of COHb concentration in blood and may be related to the apoptosis of nerve cells caused by CO poisoning. Therefore, the treatment course for the eye clinical manifestations caused by CO poisoning is rather long, and the treatment effect is poorly satisfied.

In this study, there were some patients with complaints and ophthalmic examination abnormalities, and the proportion of ophthalmic examination abnormalities was much higher than those with complaints. Despite the positive treatment, there are still some patients with unsatisfactory sequelae. Moreover, it is very important to carry out more detailed ophthalmic examinations and protect visual function for each patient with acute CO poisoning. The irreversible permanent damage may occur if it is not found in time and intervened as early as possible.

Due to the great limitation of obtaining fresh brain tissue and visual pathway specimens in the clinic, it is difficult to define the mechanism of brain tissue and visual pathway damage and repair. Scholars around the world only infer on the basis of auxiliary diagnostic technology and animal experiments. Visual evoked potential (VEP) reflects the transmission process of visual information from the retina to the visual center of the cerebral cortex. It is the electrical activity of nerve impulse from ganglion cell synapse, axon, and optic nerve to the visual cortex in the occipital lobe after the retina is stimulated by light or image. It can sensitively reflect the integrity and functional state of axon and myelin sheath of neurons in each area of the optic nerve and is considered as an objective index in the evaluation of vision road function. FVEP and pattern visual evoked potential (PVEP) are most commonly used in the clinic. At present, it is generally believed that PVEP has better stability. P100 wave is often used as the main objective evaluation index, but it is only suitable for patients with visual acuity >0.1. FVEP is the most perfect cortical evoked potential at present, and it is easy to operate. It has good effect on those who cannot cooperate with visual acuity <0.1, infants, and coma patients; especially for critically ill patients, most of them can only choose FVEP for examination and analysis. Many studies have shown that P-wave latency represents the transmission time of visual impulse in the visual pathway. The prolonged P-wave latency reflects the damage degree of the optic nerve myelin sheath and may also be related to the development of optic nerve demyelination, while the amplitude of P-wave represents the number of nerve fibers involved in visual excitation, and the change of P amplitude reflects the degree of optic nerve axon injury. In the present study, we found that there were some patients with abnormal flash visual evoked potential in three groups. Patients in the moderate poisoning group had slight abnormal P1-wave latency and amplitude, while those in the delayed encephalopathy group had severe abnormal P1-wave latency and amplitude. After statistical analysis, there was no statistical difference among the three groups. This may be due to the unstable waveform of FVEP, high variability, and easy to be affected by the external environment and the condition of the examinee. Therefore, we should pay attention to the combination of medical history and other auxiliary examination results for comprehensive consideration and specific analysis in clinical practice. At the same time, we should pay more attention to the comparison of eye changes between patients and normal people.

It is noteworthy that the incidence rate of visual impairment in patients with delayed encephalopathy is not uncommon compared with that in the acute moderate and severe poisoning groups. The clinical symptoms in the DECAMP group lasted for a long time, and the clinical treatment effect was not satisfied despite active medical help. The main reason is that CO poisoning often causes the degradation of unsaturated fatty acids, and a wide range of secondary demyelinating changes was found in the subcortical white matter, including in the lateral geniculate body. The pathological manifestations are diffuse damage of white matter and dysfunction of upper and lower conduction tracts. Clinically, there are abnormalities of motor, sensory, and reticular ascending systems, as well as delayed visual disorders in ophthalmology [4]. This is another important reason for the long course of visual dysfunction and the unsatisfactory therapeutic effect. Some patients may be combined with oculomotor nerve and optic nerve damage, or keratopathy, glaucoma, etc., which may further aggravate the clinical symptoms.

In summary, the incidence of visual dysfunction in patients with CO poisoning was high, the clinical symptoms were rich and diverse, the duration of disease was long, and the prognosis was poor. Thus, the routine ophthalmological examination and treatment should be perfected as soon as possible in clinical practice.

## Figures and Tables

**Table 1:** General information of three groups of patients.

	MP group	SP group	DE group	*F*/*χ*^2^ value	*P* value
*n*	193	165	78	—	—
Gender (M/F)	99/94	86/79	41/37	0.045	*P* > 0.05
Age (years)	56.8 ± 7.1	56.3 ± 7.3	57.2 ± 8.4	0.003	*P* > 0.05
BMI (kg/m^2^)	21.6 ± 4.2	22.4 ± 3.9	21.7 ± 4.6	1.340	*P* > 0.05
Education (years)	10.8 ± 4.2	10.5 ± 4.5	11.3 ± 3.6	0.822	*P* > 0.05

M: male; F: female; BMI: body mass index.

**Table 2:** Sequence parameters of brain MRI.

Sequence name	Weighted data	Position	TR (ms)	TE (ms)	Layer thickness (mm)	Spacing (mm)	Remarks
FSE plain scan	T2WI	Transverse	4000	95.8	5	1	Whole brain
	T2flair	Transverse	8602	146.7	5	1	Whole brain
	T1flair	Transverse	1843	20.7	5	1	Whole brain

STIR sequence	T2WI	Coronal	6000	42	5	1	Whole brain
	T1WI	Sagittalia	460	16	5	1	Whole brain
		Coronal	540	9	5	1	Whole brain

**Table 3:** Comparison of the causes of visual dysfunction in each group (Fisher exact probability method).

	MP group (*n* = 193)	SP group (*n* = 165)	DE group (*n* = 78)	*P* value
Keratopathy	3	2	0	0.848
Glaucoma	2	1	0	1.000
Optic neuropathy	2	2	1	1.000
Retinopathy	2	2	2	0.573
Oculomotor neuropathy	2	2	0	1.000
Vitreous diseases	1	2	0	0.777
Maculopathy	2	1	1	1.000
Intracranial lesions	2	2	2	0.573
Trauma	4	3	1	1.000
Hysteria	0	1	0	0.557

**Table 4:** Consistency comparison between the complaint symptom and the ophthalmic examination (Fisher exact probability).

		MP group (complaint symptom)		SP group (complaint symptom)		DE group (complaint symptom)	
		Yes (*n*)	No (*n*)	Yes (*n*)	No (*n*)	Yes (*n*)	No (*n*)
Ophthalmic examination	Normal (*n*)	2	173	1	147	1	72
	Abnormal (*n*)	10	8	9	8	1	4
*χ* ^2^ value		73.803		64.271			
*P* value		<0.01		<0.01		0.125	

**Table 5:** The analysis of flash visual evoked potential (FVEP) examination in three groups (Fisher exact probability).

	Latency of P1			Amplitude of P1		
	Mild abnormality	Moderate abnormality	Severe abnormality	Mild abnormality	Moderate abnormality	Severe abnormality
MP group (*n* = 193)	4	1	1	4	2	0
SP group (*n* = 165)	1	2	2	1	3	1
DE group (*n* = 78)	0	1	2	0	1	2
*P* value	0.358			0.181		

**Table 6:** Analysis of clinical symptoms, special ophthalmic examination, and brain MRI examination results of three groups of patients.

		MP group (*n* = 193)	SP group (*n* = 165)	DE group (*n* = 78)	*χ* ^2^ value	*P* value
Complaint symptom		12	10	2	1.583	0.453
Ophthalmic examination abnormality	Vision and visual field	9	8	3	0.126	0.938
	Fundus examination	12	10	4	0.122	0.940
	FVEP	6	5	3	0.125	0.939
Brain MRI abnormality		18	21	51	116.717	0.000

**Table 7:** Comparison of curative effect in the three groups after normal treatment (Kruskal–Wallis *H* test and Nemenyi test).

	Complete recovery (*n*, %)	Improvement (*n*, %)	Nonhealing and sequelae (*n*, %)
MP group	10/18, 55.6%	5/18, 27.8%	3/18, 16.7%
SP group	3/17, 17.6%	4/17, 23.5%	10/17, 58.9%
DE group	1/5, 20.0%	0/5, 0.0%	4/5, 80.0%
*H*_c_ value	9.218		
*P* value	0.006		

Note: Nemenyi test: MP group *vs.* SP group: *χ*^2^ = 7.10, *P*=0.03; MP group *vs.* DE group: *χ*^2^ = 4.98, *P*=0.08; for SP group *vs.* DE group: *χ*^2^ = 0.20, *P*=0.91.

**Table 8:** Score comparison of the NEI-VFQ-25 in the three groups in two months after treatment.

	MP group (*n* = 193)	SP group (*n* = 165)	DE group (*n* = 78)	*F* value	*P* value
General health	74.59 ± 8.12	72.10 ± 10.35	62.31 ± 16.87	35.242	*P* < 0.05
General vision	79.02 ± 10.96	75.90 ± 11.44	68.31 ± 14.53	22.697	*P* < 0.05
Ocular pain	86.22 ± 5.35	85.15 ± 6.95	86.02 ± 4.32	1.560	*P* > 0.05
Near activities	75.28 ± 10.59	68.82 ± 13.25	61.93 ± 17.60	30.940	*P* < 0.05
Distance activities	75.43 ± 10.40	70.55 ± 11.51	62.27 ± 16.41	33.335	*P* < 0.05
Social function	80.96 ± 9.02	77.53 ± 10.06	65.25 ± 18.47	51.191	*P* < 0.05
Mental health	76.82 ± 11.41	73.70 ± 10.53	56.17 ± 17.75	78.680	*P* < 0.05
Role difficulties	79.53 ± 11.82	75.74 ± 13.05	52.29 ± 17.90	116.929	*P* < 0.05
Dependency	82.16 ± 8.43	77.42 ± 11.51	62.06 ± 18.38	79.483	*P* < 0.05
Driving	63.03 ± 5.52	62.52 ± 7.67	61.93 ± 9.58	0.684	*P* > 0.05
Color vision	93.47 ± 4.59	92.81 ± 6.37	92.54 ± 9.23	0.794	*P* > 0.05
Peripheral vision	81.83 ± 9.94	76.57 ± 10.43	69.60 ± 15.69	33.441	*P* < 0.05

## Data Availability

The data used to support the findings of the study are available from the corresponding author on request.
