# Corrigendum to “Clinical Characteristics of Visual Dysfunction in Carbon Monoxide Poisoning Patients”

**DOI:** 10.1155/2021/6287083

**Published:** 2021-01-16

**Authors:** Wei-Kang Bi, Jing-Lin Wang, Xu-Dong Zhou, Ze-Kun Li, Wen-Wen Jiang, Shao-Bin Zhang, Yong Zou, Ming-Jun Bi, Qin Li

**Affiliations:** ^1^Weifang Medical University, Weifang, Shandong 261042, China; ^2^Department of Integration of Chinese and Western Medicine, Yantai Yuhuangding Hospital Affiliated to Qingdao University, Yantai, Shandong 264000, China; ^3^Emergency Center, Yuhuangding Hospital Affiliated to Qingdao University, Yantai, Shandong 264000, China; ^4^Shandong Wendeng Osteopathic Hospital, Yantai, Shandong 264000, China; ^5^Centre of Integrated Chinese and Western Medicine, School of Basic Medicine, Qingdao University, Qingdao, Shandong 266071, China; ^6^Weifang Eye Hospital, Weifang, Shandong 261000, China

In the article titled “Clinical Characteristics of Visual Dysfunction in Carbon Monoxide Poisoning Patients” [1], there was a spelling error in author Jing-Lin Wang's name in the author list, where “Jin-Lin Wang” should have read as “Jing-Lin Wang.” This is corrected in the author list above.
